# Prognostication of outcome after cardiac arrest and targeted temperature management

**DOI:** 10.1186/1471-227X-15-S1-A10

**Published:** 2015-06-24

**Authors:** Christoph Leithner

**Affiliations:** 1 Department of Neurology, Charité-Universitätsmedizin, Berlin, Germany

## 

The vast majority of cardiac arrest (CA) survivors remain comatose at hospital arrival. Around one-half of these patients suffer from severe hypoxic encephalopathy leading to death or unresponsive wakefulness. Early outcome prognostication may reduce futile intensive care and suffering for next of kin. Several early prognostic markers have shown high positive predictive value for poor outcome in patients treated at normothermia, but results may be altered by targeted temperature management (TTM).

Most prominently, absent motor response to painful stimuli 3 days after CA and a serum concentration of neuron-specific enolase (NSE) above 33 ng/ml do not predict poor outcome reliably in these patients [1,2]. Sedation during TTM is a relevant confounder for motor response and corneal reflexes [3]. Bilaterally absent pupillary light reaction 3 days after CA is a reliable predictor of poor outcome (sensitivity 20%) [1]. Eye diseases and medication are potential confounders.

A threshold for poor outcome prediction by NSE has not been established after TTM. Above a level of 80 to 100 ng/ml 72 hours after CA good outcome has rarely been reported. The NSE levels may vary with different test kits, and NSE producing tumors, acute brain diseases and severe hematological diseases/hemolysis are confounders.

Bilateral absence of cortical median nerve SSEP remains a reliable predictor, but single cases with recovery were reported [4] and inter-rater reliability is not perfect. Preserved spinal SSEP and low cortical noise levels are important prerequisites. The sensitivity of absent SSEP for poor outcome prediction is around 40% [1]. EEG can be a valuable predictive tool when interpreted by experienced neurophysiologists and indicates (subclinical) status epilepticus [5]. The influence of medication on EEG needs to be considered. Recently, a revised EEG classification has been proposed [6].

A reduced contrast between gray and white matter in brain computed tomography is associated with poor outcome. It can be quantified as gray–white-matter ratio and values below 1.1 predict poor outcome with high specificity [7]. Additional studies are needed to establish this threshold more firmly. MRI emerges as a prognostic parameter because of high spatial resolution and high sensitivity for cytotoxic brain edema with DWI/ADC imaging [8]. However, access is limited and quantification of early changes needs to be established in larger cohorts.

To date, most authors argue for a multiparameter prognostication approach including repeated neurological examination, SSEP, NSE, EEG and imaging (CT or MRI) combined with a waiting period for potential recovery of several days after cardiac arrest.

## Financial disclosure

CL has received honorarium and/or travel costs from C. R. BARD.

## Declaration

This abstract and supplement was proposed, developed and commissioned by BMC Emergency Medicine and was funded by an educational grant from C. R. BARD, NJ, USA. The published abstract was independently prepared by the author. C. R. BARD had no input into the content.
